# Trends in Selective Internal Radiation Therapy (SIRT) for Treating Hepatocellular Carcinoma, Cholangiocarcinoma, and Liver Metastasis: A Total Population Analysis from 2006 to 2021 in Germany

**DOI:** 10.3390/curroncol30120752

**Published:** 2023-12-05

**Authors:** Smita George Thoduka, Luka Flegar, Christer Groeben, Johannes Huber, Nicole Eisenmenger, Tobias Paulus, Katharina Vogt, Markus Luster, Nasreddin Abolmaali

**Affiliations:** 1 Department of Nuclear Medicine, Philipps University of Marburg, 35043 Marburg, Germany; luster@med.uni-marburg.de; 2 Department of Urology, Philipps University of Marburg, 35043 Marburg, Germany; luka.flegar@uk-gm.de (L.F.); christer.groeben@uk-gm.de (C.G.); johannes.huber@uk-gm.de (J.H.); 3 Reimbursement Institute, 30354 Hürth, Germany; ne@reimbursement.institute; 4 Department for Diagnostic and Interventional Radiology and Nuclear Medicine, St. Josef Hospital, Ruhr-University Bochum, 44791 Bochum, Germany; tobias.paulus@klinikum-bochum.de (T.P.); nasreddin.abolmaali@kklbo.de (N.A.); 5 Department of Radiology and Interventional Radiology, University Hospital Freiburg, 79110 Breisgau, Germany; katharina.vogt@uniklinik-freiburg.de

**Keywords:** SIRT, population-based, trends, hepatocellular carcinoma (HCC), cholangiocarcinoma (CCC), liver metastasis

## Abstract

The aim of this study was to investigate trends in selective internal radiation therapy (SIRT) for hepatocellular carcinoma (HCC), cholangiocarcinoma (CCC), and liver metastasis in Germany. We analyzed the nationwide German hospital billing database from 2006 to 2019 for the diagnosis of HCC, CCC or liver metastasis in combination with SIRT. For analyses of SIRT on the hospital level, we used the reimbursement.INFO tool based on German hospitals’ quality reports from 2008 to 2021. Linear regression analysis was performed to detect changes over time. We included a total of 14,165 SIRT procedures. The annual numbers increased from 99 in 2006 to 1605 in 2015 (*p* < 0.001; increase by 1521%), decreasing to 1175 cases in 2019 (*p* < 0.001). In 2008, 6 of 21 hospitals (28.6%) performed more than 20 SIRTs per year, which increased to 19 of 53 (35.8%) in 2021. The share of SIRT for HCC increased from 29.8% in 2006 to 44.7% in 2019 (*p* < 0.001) and for CCC from 0% in 2006 to 9.5% in 2019 (*p* < 0.001), while the share of SIRT for liver metastasis decreased from 70.2% in 2006 to 45.7% in 2019 (*p* < 0.001). In-hospital mortality was 0.2% after the SIRT procedure. Gastritis (2.7%), liver failure (0.4%), and sepsis (0.3%) were the most common in-hospital complications reported. We observed an increase in SIRT procedures in Germany, with the number of hospitals offering the procedure going up from 21 in 2008 to 53 in 2021. While the treatment of liver metastasis remains the most common indication, SIRT for HCC and CCC increased significantly over the last few years. The mortality and complication rates show that SIRT is a relatively safe procedure.

## 1. Introduction

A large majority of patients with primary or secondary hepatic malignancies do not qualify for surgical or percutaneous treatments. Even systemic treatments, such as chemotherapy, immunotherapy, or biological therapy are limited in patients who experience early recurrence or low response, as well as in those experiencing significant side effects or intolerance to the therapy. These patients benefit from transarterial therapies, such as transarterial embolization (TAE) or chemoembolization (TACE), and in some cases, radioembolization (TARE), also known as selective internal radiation therapy (SIRT) [1]. These interventional radiology therapies utilize the differing blood supply of malignant tissue over the hepatic arteries compared with that of normal healthy liver tissue over the portal vein [2,3]. These treatments are often performed to control the disease and as bridging therapy to prepare the patient for potential surgical or local ablative therapies [4,5].

Among the transarterial therapies, studies comparing TACE and SIRT have demonstrated that single-session SIRT is as effective as multiple sessions of TACE, especially in the treatment of HCC, while having significantly fewer complications [6]. Furthermore, SIRT remains a treatment option for patients with portal vein thrombosis, which is a relative contraindication for TAE and TACE [7]. SIRT has been shown to be a safe and effective treatment with the option of personalized dosimetry, which has been proven to improve response rates and overall survival. The decision to use SIRT is made by a multidisciplinary tumor board, and the planning and optimization of SIRT are achieved through close cooperation between radiology and nuclear medicine [3].

The most commonly used radionuclide in SIRT has been Yttrium-90-labeled resin (SIRTeX) or glass (Therasphere) microspheres. Other radionuclides used have been Holmium-166 (QuiremSpheres) and Rhenium-188. SIRT acquired FDA approval in the US in 2002 and has been available in Europe since 2003 when SIRTeX received European approval [8].

The recently published study by Radosa et al. showed the high adoption of interventional oncology in Germany, with 216 radiology clinics in Germany offering minimally invasive oncological therapies in the year 2019 [9]. Our aim was to study the availability of SIRT as a treatment option in Germany and track the trend from 2006 to 2021.

## 2. Materials and Methods

### 2.1. Database

Data from German hospitals’ quality reports and from the German Billing Database (Destatis) were studied. The Destatis database was used for the analysis of all SIRT procedures, while the German hospitals’ quality reports were used to identify national providers of SIRT. Table 1 provides an overview of the different databases. Identification of the patient cohort, as well as the data extraction method, have previously been described by our working group investigating patients with muscle-invasive bladder cancer [10].

### 2.2. German Billing Database (Destatis)

The German Federal Statistical Office (Destatis) has collected reimbursement data of inpatient treatment since 2004. German hospitals are legally obliged to transmit these data to Destatis, which is why this database represents a total population sample. Patients with a diagnosis of HCC (ICD code “C22.0”), CCC (ICD code “C22.1”) or liver metastasis (ICD code “C78.7”) in combination with SIRT were included. The OPS code for SIRT changed several times during our study period. In 2006, code “8-530.44” was used for SIRT. Between 2007 and 2011, the code “8-530.45” represented SIRT. Since 2012, the code “8-530.a5” has been used for the coding of SIRT. We further analyzed specific SIRT approaches with Rhenium-188-labeled microspheres (from 2010 to 2011, OPS code “8-530.48”; from 2012, OPS code “8-530.a6”) and Holmium-166-labeled microspheres (OPS code “8-530.a8”) in combination with the ICD code “C22.0”, “C22.1”, and “C78.7”. Additionally, we analyzed the length of hospital stay (LOS), in-hospital mortality, caseload distribution (hospitals with <20, 20–49, and >50 cases per year), as well as blood transfusion after SIRT. Furthermore, to detect the most common complications associated with SIRT, we included the ICD codes “R58” acute bleeding, “K72” liver failure, “K81” cholecystitis, “K29” gastritis, “K29.8” duodenitis, “N17” acute renal failure, and “A02, A20, A26, A32, A40, A41, A42, B37” for sepsis.

### 2.3. German Hospitals’ Quality Reports

The reimbursement.INFO tool (Reimbursement Institute, Hürth, Germany), based on billing data from hospitals’ quality reports, was used to analyze SIRT on an institutional level. We used the OPS codes “8-530.a5”, “8-530.a6”, and “8-530.a8”. Map displays were created using “EasyMap 11.1 Standard Edition” software (Lutum+Tappert DV-Beratung GmbH, Bonn, Germany).

### 2.4. Statistical Analysis

Data are presented as absolute and relative frequencies. For the detection of trends over time, we implemented linear regression models. Statistical significance was defined by *p* < 0.05. We used SPSS 28.0.1. (IBM corp., Armonk, NY, USA) for the present statistical analysis.

### 2.5. Ethics Statement

The data presented in this study were obtained in accordance with the World Health Association Declaration of Helsinki in its latest version. Analyzed data were completely anonymized and derived from established databases with rigorous data protection measures. Therefore, an additional ethics statement, as well as a written consent, were not required.

## 3. Results

**Figure 1:**
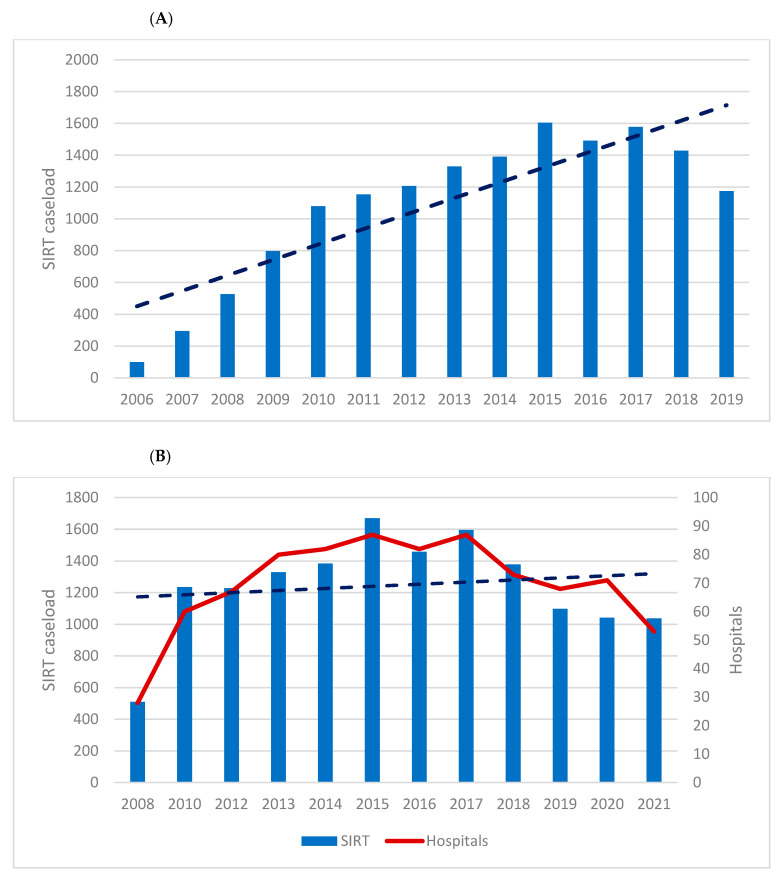
Total case numbers of SIRT from 2006 to 2021 in Germany are represented by the blue columns. The red line represents the hospitals offering the therapy. The dotted line represents the trend (source: (**A**) Destatis and (**B**) German hospitals’ quality reports).

In total, 14,165 SIRT cases were included in the present analysis. Figure 1 displays the case numbers of performed SIRT from 2006 to 2019. The annual numbers increased from 99 in 2006 to 1605 in 2015 (*p* < 0.001; increase of 1521%), and subsequently decreased slightly to 1175 cases in 2019 (*p* < 0.001).

**Figure 2:**
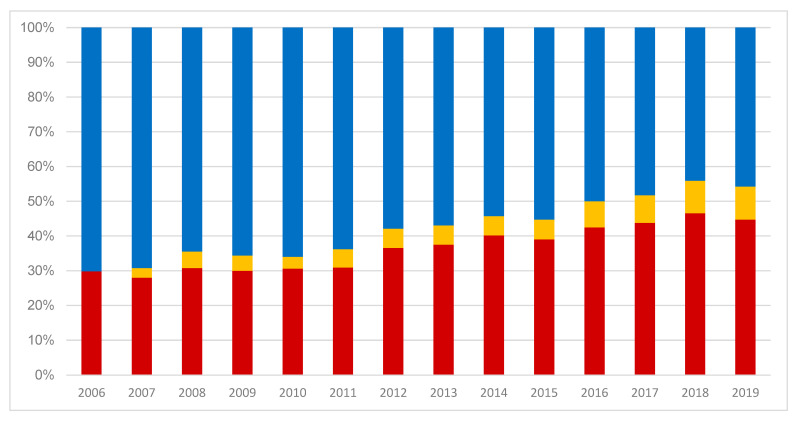
SIRT in Germany: distribution for treatment indication of HCC (red), CCC (orange), and liver metastasis (blue) between 2006 and 2019 (source: Destatis).

Figure 2 demonstrates the distribution of SIRT treatments for HCC, CCC, and liver metastasis over the same time period. The share of SIRT for HCC increased significantly from 29.8% in 2006 to 44.7% in 2019 (*p* < 0.001). The share of SIRT for CCC increased from 0% in 2006 to 9.5% in 2019, while that for liver metastasis decreased from 70.2% in 2006 to 45.7% in 2019 (*p* < 0.001). Absolute numbers are presented in Supplementary Materials Figure S1.

**Figure 3:**
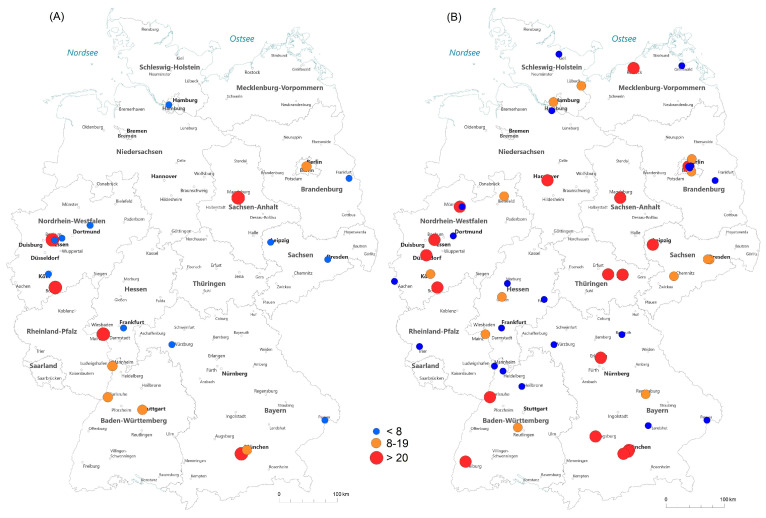
Overview of hospitals performing SIRT in Germany in 2008 (**A**) and 2021 (**B**) (source: German hospitals’ quality reports).

Figure 3 gives an overview of hospitals in Germany performing SIRT in 2008 and 2021. In 2008, 6 of 21 hospitals (28.6%) performed more than 20 SIRTs per year, which increased to 19 of 53 (35.8%) in 2021. In 2021, 13 hospitals (24.5%) performed 5–19 SIRTs and 21 hospitals (39.6%) hospitals performed <8 SIRTs. In 2008, 47.6% (10/21) of institutions offering SIRT were university hospitals, which increased to 62.2% (33/53) in 2021.

In 2021, 62.5% of treated patients with SIRT were male (female 37.5%) and 55.2% of all treated patients (female and male) were over 65 years of age.

In-hospital mortality was 0.2% after the SIRT procedure (28 out of 13,981), with varying mortality rates observed for different clinical diagnoses. In total, 8/5491 patients died after SIRT for HCC, 12/905 patients died with SIRT for CCC, and 8/7585 patients died after SIRT for liver metastasis.

The overall LOS after SIRT in Germany decreased from a median of 4 days (SD: 6.5 days) in 2006 to 3 days (SD: 3.2 days) in 2019 (*p* < 0.001). Clinical diagnosis for SIRT had no significant influence on LOS.

The overall rate of blood transfusion in Germany after SIRT treatment was 1.4% (191 out of 13,981). Blood transfusions were administered more commonly after SIRT for liver metastasis (1.6%) vs. CCC (1.0%) treatment by SIRT vs. 0.9% after SIRT for HCC.

The most common in-hospital complications associated with SIRT were gastritis in 2.7%, liver failure in 0.4%, and sepsis in 0.3%. Acute kidney failure occurred in 0.3%, duodenitis in 0.2%, and cholecystitis in 0.1%. Acute bleeding was reported in 0.02% of cases.

**Figure 4:**
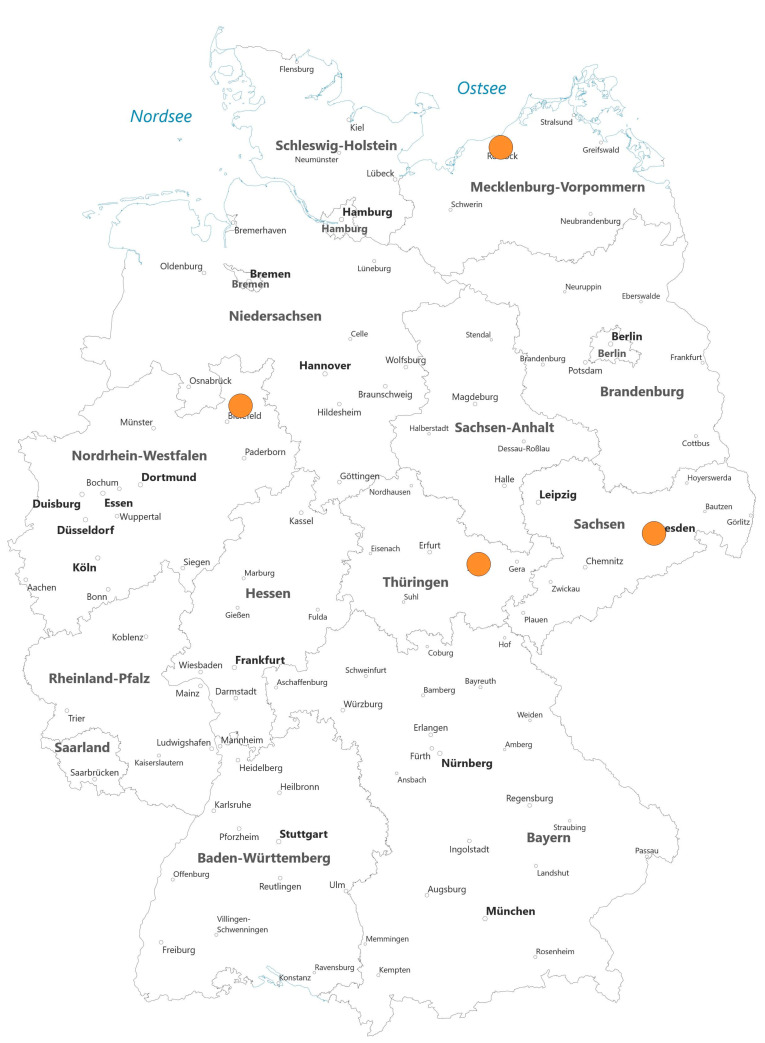
Overview of hospitals performing SIRT with Holmium in Germany in 2021 (source: German hospitals’ quality reports).

The use of Holmium-166-labeled microspheres increased from one hospital offering the therapy in 2016 to four hospitals in 2021, with up to 45 procedures (Figure 4). Furthermore, three German hospitals used Rhenium-188-labeled microspheres, performing a total of six procedures between 2012 and 2021.

## 4. Discussion

The present population-based study showed that SIRT cases increased from a total of 99 cases in 2006 to 1175 cases in 2019 (*p* < 0.001). In 2021, 53 German hospitals offered SIRT for treatment of HCC, CCC, and liver metastasis. A recently published study by Krieg et al. investigating TACE for liver tumors in Germany showed similar increasing trends in TACE [11].

### 4.1. HCC, CCC, and Liver Metastasis

SIRT demonstrated a general increase across all studied indications, with the treatment of HCC showing the most substantial increase in share. This may be attributed to SIRT’s potential for curative intent in HCC, as well as its efficacy as a bridging therapy to curative surgical approaches. Notably, Cucchetti et al. proposed that SIRT could significantly reduce tumor size in unresectable hepatocellular and cholangiocellular carcinomas, potentially enabling resection [4]. Radiation segmentectomy, especially, has been shown to have promising results for primary and secondary liver malignancies limited to one segment. A recent systematic review investigating oncologic outcomes after radiation segmentectomy demonstrated favorable tumor control, with outcomes similar to ablation and surgical resection in patients with tumors up to 3 cm being reported [12,13]. In comparison to TACE, radiation segmentectomy has been shown to improve time-to-progression, complete response rate, as well as local tumor control [13]. Radiation segmentectomy with SIRT can be curative, and therefore, might have been the reason for the popularity of Yttrium-90 SIRT in recent years. Additionally, the combination of SIRT with immunotherapy has shown promising outcomes [14]. Furthermore, in 2018, the ESMO Clinical Practice Guidelines for the diagnosis, treatment, and follow-up of HCC have integrated and recommended SIRT for intermediate Stage HCC [15]. Recently, SIRT was recommended for early-stage disease by the Barcelona Clinic Liver Cancer (BCLC) 2022 guideline update. Studies showed that radiation segmentectomy achieved complete pathologic necrosis in BCLC 0-A HCC, making it attractive for patients who are unfit for surgery or ablation [16]. Unfortunately, in 2019, the caseload experienced a decline, mainly attributed to the disruptive effects of the COVID-19 pandemic.

The treatment of CCC by SIRT has also increased. Edeline et al. described solid results for the combination of chemotherapy and SIRT as first-line treatment of unresectable CCC. The authors showed that a significant proportion of patients were downstaged to surgical intervention [17].

The share of liver metastasis mainly due to colorectal carcinoma (CRC) treated with SIRT has decreased. This might be due to the fact that through advances in immunotherapy, chemotherapy refractory or intolerant metastatic colorectal carcinoma, which were previously treated by SIRT, might nowadays be treated with immune checkpoint inhibitors. However, our results showed that SIRT was still performed in 46% of patients with a secondary hepatic malignancy in 2019.

### 4.2. Patient’s Age, Caseload Distribution, and University Setting

The present analysis shows, that more than half of the SIRT treatments performed in Germany in the studied years had been in patients over the age of 65 years. Numerous studies have consistently shown that age is not a predictive factor for adverse effects of SIRT in patients with HCC or liver metastases of CRC, and tumor efficacy remains unaffected by age. Consequently, age should not be regarded as a limiting factor when making decisions regarding the use of SIRT [18].

We noticed a trend towards the centralization of SIRT, with one out of three patients being treated in a high-volume hospital. Furthermore, 62% of clinics offering SIRT were university hospitals in 2021. Several studies showed a better outcome for patients treated in high-volume hospitals with lower morbidity and fewer side effects [10,19].

### 4.3. In-Hospital Complications and Mortality, Blood Transfusions, and LOS

Gastritis (2.7% of cases), liver failure (0.4% of cases), and sepsis (0.3% of cases) were the most common in-hospital complications reported. Murthy et al. presented a similar low incidence of gastrointestinal complications [20]. Several studies have shown the importance of careful patient selection in avoiding common complications [21].

In-hospital mortality was 0.2% after the SIRT procedure in the present analysis, with similar results shown by Mertens et al. [22]. Several studies have shown that SIRT is a safe procedure. A study from 2017 concluded that SIRT appears to be safely integrated into the standard-of-care pathways for most tumor types [23]. Blood transfusions were required in 1.4% of cases after SIRT. LOS after SIRT in Germany decreased from 4 to a median of 3 days in 2019, and clinical diagnosis did not significantly affect LOS.

### 4.4. Holmium, Rhenium, and Yttrium

The most frequently utilized radionuclide in SIRT is Yttrium-90, which is a ß* emitter with low positron emission. The current clinical procedure for dose planning involves a pre-therapeutic scan using Technetium-99m-labeled MAA, positioning the catheter similarly to the planned therapy, followed by a SPECT/CT scan to calculate the lung dosage and to estimate the dosage applied to the tumor compared with normal liver tissue [24].

Standardized models enable personalized dosimetry of Y90 [25]. After SIRT, a PET/CT scan utilizing the low positron emission from Y90 can be employed to compute the absorbed dose to the tumor relative to the liver tissue [24,25]. In the present analysis, we were able to show that most SIRTs were performed with Yttrium-90.

The use of Holmium-166-labeled microspheres has been on the rise in Germany. In 2016, only one hospital offered this therapy, whereas in 2021, four clinics performed up to 45 procedures. Holmium-166 dosimetry can be conducted using MR imaging in addition to classic SPECT dosimetry due to its paramagnetic properties [26]. Furthermore, the planning procedure involves the utilization of the same microspheres with identical flow dynamics to those used during the therapy. This approach has been demonstrated to improve dosimetry through the application of standardized models [27,28].

Only three hospitals in Germany have been known to offer SIRT using Rhenium-188-labeled microspheres, with a total of six procedures in total performed in the years 2012 to 2021. Shukla et al. discussed 188Re-based therapy as a cost-effective and more affordable option when compared with other available therapeutic radioisotopes [29].

### 4.5. Limitations and Strengths

The present study has several limitations. First, our queried databases do not provide clinical information such as tumor stage or size. Furthermore, we were able to only cover in-hospital treatments. However, due to legal regulations in Germany, a minimum 2-day inpatient stay in a nuclear medicine therapy ward is mandatory after SIRT procedure. The code for SIRT changed a few times during our analyzed study period, which may have led to selection bias. While the effectiveness of SIRT in handling primary and secondary hepatic tumors has been studied, the analysis of trends within Germany can lead to a better understanding of the adoption of this therapy. The steady increase in the number of clinics offering this treatment option, combined with an increase in the number of procedures carried out, demonstrates the acceptance among clinicians as well as patients for this innovative therapy. With an increase in the number of procedures, further optimization of the procedure and planning can improve patient outcomes. The close cooperation of radiologist and specialists of nuclear medicine along with surgeons and oncologists can improve patient selection and also provide a more individualized therapy for the benefit of the patient.

## 5. Conclusions

We have observed an increase in SIRT in Germany. While the treatment of liver metastasis is the most common indication, SIRT for HCC and CCC has increased significantly over the last years. Furthermore, the present study showed that mortality and complication rates were low and SIRT is a safe procedure. Recent updates of the BCLC 2022 guideline have confirmed the relevance of SIRT in the treatment of primary and secondary liver malignancies.

## Figures and Tables

**Table 1:** Summary of the queried databases.

Source of data	Nationwide hospital billing database of the German Federal Statistical Office (Destatis database)	German hospitals’ quality reports (reimbursement.INFO tool)
Details	Age and gender Diagnosis code in combination with OPS code Surgical access route Hospital characteristics (teaching status, size, annual surgery/procedure caseload, approaches for surgery/procedure)	Age and gender Diagnosis code or OPS code (no combination possible) Hospital characteristics (teaching status, annual surgery/procedure caseload) Geographical localization of respective hospitals
Number of patients	13,981	14,165
Proportion of population	100%	100%
Years	2006–2019	2008–2021 (missing: 2006, 2007, 2009, 2011)

## Data Availability

All datasets used in this study are stored centrally at the specific institutes (German Federal Statistical Office—Destatis; German Quality reports) and are available on request from the corresponding author.

## Supplementary Materials

The following supporting information can be downloaded at: https://www.mdpi.com/article/10.3390/curroncol30120752/s1, Figure S1: Absolute numbers of SIRT procedures in Germany for HCC (red), CCC (orange), and liver metastasis (blue) between 2006 to 2019 (source: Destatis).
