# Supplementary material for: Trends in Selective Internal Radiation Therapy (SIRT) for Treating Hepatocellular Carcinoma, Cholangiocarcinoma, and Liver Metastasis: A Total Population Analysis from 2006 to 2021 in Germany
Source: Curr Oncol. 2023 Dec 5;30(12):10325–35. doi: 10.3390/curroncol30120752 (PMC10742573; doi:10.3390/curroncol30120752)
Supplement: Supplementary file 1 [file curroncol-30-00752-s001.zip › curroncol-2671875-supplementary.pdf]

Supplementary data:

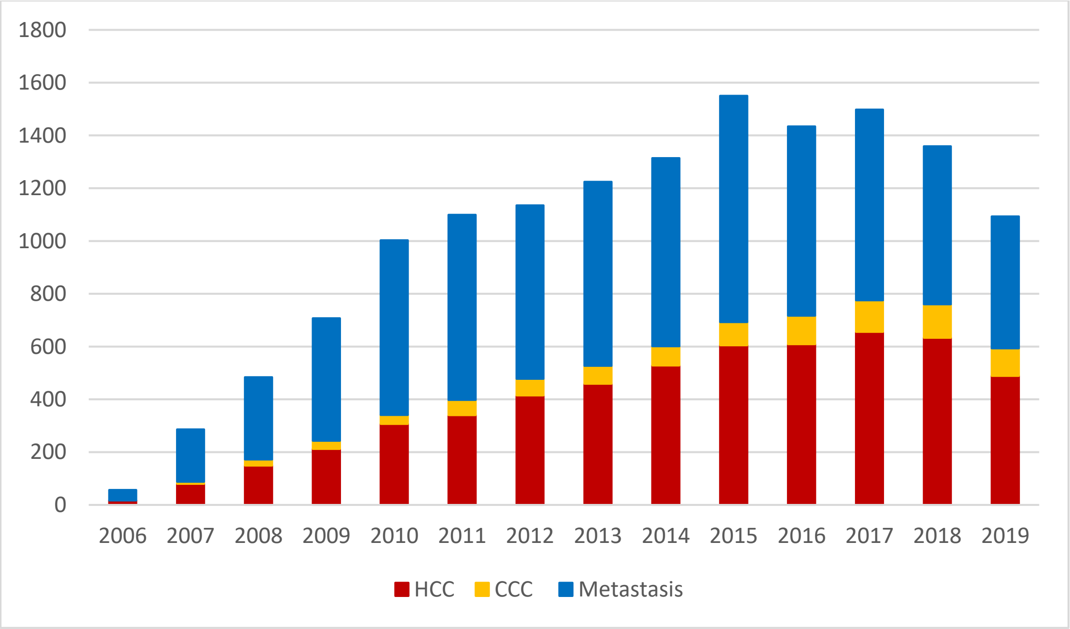

Figure S1. Absolute numbers of SIRT procedures in Germany for HCC (red), CCC (orange) and liver metastasis (blue) between 2006 to 2019 (source: Destatis).
